# Work changes and employee age, maladaptive coping expectations, and well-being: a Swedish cohort study

**DOI:** 10.1007/s00420-021-01824-6

**Published:** 2022-01-07

**Authors:** Annelies E. M. Van Vianen, Michelle Van Laethem, Constanze Leineweber, Hugo Westerlund

**Affiliations:** 1 Department of Work and Organizational Psychology, University of Amsterdam, Postbox 15919, 1001 NK Amsterdam, Netherlands; 2 Department of Psychology, Stress Research Institute, Stockholm University, Stockholm,, Sweden

**Keywords:** Aging, Maladaptive coping expectations, Reorganization, Job changes, Satisfaction, Sleep disturbances

## Abstract

**Purpose:**

Older workers are expected to suffer more from work changes than younger ones, but empirical evidence is lacking. Negative responses to work changes may result rather from maladaptive coping expectations. This study examined possible age differences in job and life satisfaction, and sleep disturbances, after work changes (voluntary and involuntary job changes, reorganizations) and the moderating role of maladaptive coping expectations.

**Methods:**

Four biennial waves from the Swedish Longitudinal Occupational Survey of Health (SLOSH) including respondents who participated in all four waves (*n* = 3084). We used multilevel path analyses to estimate direct and moderated relationships between work changes and outcomes.

**Results:**

Involuntary job changes were associated with lower job and life satisfaction and more sleep disturbances. Reorganizations were only associated with lower job satisfaction. Older employees were more satisfied with their jobs and lives than younger employees and experienced more sleep disturbances. After involuntary job changes, older employees had similar (lower) levels of well-being as younger ones, but they reported more sleep disturbances when having experienced reorganizations. Maladaptive coping expectations were related to lower job and life satisfaction and more sleep disturbances. Employees with maladaptive coping expectations reported more sleep disturbances after involuntary job changes and reorganizations.

**Conclusion:**

Our results suggest that there are few age differences in well-being after work changes. Employee well-being seems to mostly depend on maladaptive coping expectations. Organizations aiming to prepare employees for job changes and reorganizations could focus their efforts on employees with maladaptive expectations rather than on older ones.

## Introduction

Due to globalization, competition and technological progress, jobs and organizations change rapidly, which challenges employees to prepare for and adapt to job transitions and reorganizations (Savickas et al. 2009). In times of change, the skills and jobs of older workers (> 50 years) are particularly at risk of obsolescence, requiring them to cope with work changes they may not have wanted (OECD 2019). Work changes may cause resistance, distress, and lower well-being in employees, especially when these are imposed on them, such as involuntary job changes and reorganizations (Chadi and Hetschko 2018; Fløvik et al. 2019; Jensen et al. 2019). Older workers are often expected to prefer keeping their work routines and to suffer more from work changes than younger employees do (Van Dalen et al. 2010; Van Vianen et al. 2011). The question is whether this expectation is based on actual experiences with, or rather stereotypic ideas about, older workers. Organizations may experience in practice that older workers respond negatively to changes (i.e., complaints, dissatisfaction) resulting in their lower well-being during and after the change. Because older workers constitute an increasing part of the labor force in most European countries (OECD 2020), these adverse responses would be a serious threat to the performance and innovative strength of organizations. Moreover, low job satisfaction could push workers toward earlier retirement (Davies et al 2017; Zacher and Rudolph 2017), which increases labor shortage (Sullivan and Ariss 2019).

It is conceivable that older workers may be harmed more by job changes and reorganizations than younger workers. First, older workers are on average longer tenured and when being in a job for a long time it can be more difficult to unlearn routinized skills and work procedures (Niessen et al. 2010). Second, older workers tend to be less involved in training activities (Boockmann et al. 2018, OECD 2019) and they may associate work changes with a loss of earned privileges and status (Yeatts et al. 2000). For example, reorganizations may raise job insecurity in older workers as older workers tend to have more difficulties finding reemployment (Wanberg et al. 2016). Additionally, older workers have a shorter work future and may perceive less opportunities for accomplishing goals (e.g., modifying their work or tasks) in the work period up to their retirement (Zacher and Frese 2009). Moreover, Socioemotional Selectivity Theory (SST) (Carstensen 2006) suggests that a shorter (rather than open) time perspective prompts the selection of emotion-related (rather than knowledge-related) goals in older employees and a focus on attaining positive work experiences and avoiding negative ones (Truxillo et al. 2015). Finally, life span development theories, such as Selection, Optimization, and Compensation (SOC) theory, suggest that motives related to maintenance and security will increase while motives related to development, learning and growth will decrease across the life span (Baltes et al. 1999). Job changes and reorganizations are at odds with increased security motives in older employees.

Despite these theoretical and plausible considerations, there is no empirical evidence that older workers are less engaged (Kim and Kang 2017) and more resistant to work changes (Ng and Feldman 2012) than younger workers. Moreover, meta-analytic research could not convincingly establish a positive relationship between age and security motives (Kooij et al 2011). All in all, we do not know how older, as compared to younger, workers respond to job changes and reorganization, because research on possible age differences in individual well-being following work changes is lacking.

The expectation that older workers will experience more difficulties with changes may not arise from reality but may originate in existing ideas on aging. That is, organizations may rely on general generational and age stereotypes assuming, for example, that aging comes with a greater need for certainty and stability, and lower adaptiveness to change (OECD 2020; Posthuma and Campion 2009). These stereotypic beliefs could lead organizations to adopt measures that protect older workers from change and development (Dalhoeven et al. 2016; Van Vianen et al 2011), which may limit the contribution of these workers to an organization’s performance (OECD 2020), or they could develop costly age-specific human resource programs aimed at facilitating the adaptation and retention of older workers (Truxillo et al 2015).

When studying the consequences of job changes on employee well-being, it is important to distinguish between voluntary and involuntary job changes (Garthe and Hasselhorn 2021). Voluntary job changes are associated with employees’ need for self-development, job improvement and career opportunities and, therefore, tend to lead to positive employee outcomes, such as higher job and life satisfaction (Chadi and Hetschko 2018, 2021). Involuntary job changes are associated with organizational reorganization, downsizing, and layoffs, which may lead to lower employee well-being. Organizational reorganization generally has acute adverse effects, such as lower job and life satisfaction, and more sleep disturbances (Bamberger et al. 2012; De Jong et al. 2016, Greubel and Kecklund 2011, Rafferty and Jimmieson 2017). However, individuals vary in how they respond to changes (Bamberger et al. 2012; Flovik et al. 2019) and reorganizations can provide employees with learning experiences that foster positive attitudes toward learning, development, and engagement, also in older employees (Dalhoeven et al. 2016).

The sparse research that directly examined the outcomes of involuntary job changes could not establish negative effects on employee well-being (Chadi and Hetschko 2021; Equeter et al. 2018; Garthe and Hasselhorn 2021). Instead, employees’ responses to involuntary job changes seem to depend on the extent to which they perceived the involuntary change as professionally and personally beneficial (Equeter et al. 2018). The more positive these perceptions, the more they reported positive outcomes. Apparently, involuntary transitions can be turned into an opportunity for professional and personal development.

All in all, individuals differ in their capacity to cope with stressful events, such as reorganizations and involuntary job changes. The Cognitive Activation Theory of Stress (CATS) (Ursin and Eriksen 2004; Meurs and Perrewé 2011) proposes that individuals’ responses to stress depend on their acquired expectancy of being able to handle the stressor. An adaptive coping expectancy will decrease stress responses, whereas a maladaptive coping expectancy (cognitions of helplessness and hopelessness) will increase stress responses. Recent research (Ejdemyr et al. 2021) has shown a significant association between maladaptive coping expectations and depression and anxiety, and somatic complaints, such as fatigue and sleep problems. Based on CATS and prior research, we hypothesized that a reorganization or involuntary job change would primarily harm the well-being of employees with maladaptive coping expectations and less, if at all, that of employees with more adaptive coping expectations. Given the lack of research examining the consequences of work changes for the well-being of older employees, we explored (rather than hypothesized) possible age difference in responses to change. To date, there is little empirical evidence that such an age difference would exist, with the exception that older workers were found to be less satisfied with a new job after a period of unemployment than younger workers (Wanberg et al. 2016). Additionally, research on employee maladaptive coping expectations (Stengård et al. 2017) did not find a significant association between these expectations and age. Moreover, older employees seem as good as or slightly better able than younger employees to regulate their emotions in times of stressful events, such as when experiencing negative work stressors (Doerwald et al. 2016; Scheibe and Zacher 2013). Conservation of Resources (COR) theory (Hobfoll 2002) argues that humans are motivated to obtain and retain resources, including emotional resources, and continue to build resources as they grow older. Indeed, age was found to be positively associated with emotion regulation (Kim and Kang 2017).

The present four-wave cohort study is the first to examine age differences in the associations between work changes (voluntary and involuntary job changes, and organizational reorganizations) and employee well-being (job and life satisfaction, and sleep disturbances), allowing testing relationships at both the within-persons and the between-persons level. We investigated employee well-being after work changes and whether well-being outcomes depended on employee age and maladaptive coping expectancies. In doing so, we respond to the call for more research considering employee characteristics that moderate the relationship between work changes and well-being outcomes, such as employee age (Flovik et al. 2019). By combining employee age and maladaptive coping expectations as moderators, we address a persistent age stereotype that older workers are less able than younger workers to adapt to potentially stressful work events. The findings of this study are important for employers to focus their human resource efforts on improving the adjustment and well-being of all workers or especially those of older workers.

## Methods

### Study design and participants

This study includes data on participants in the biennial Swedish Longitudinal Occupational Survey of Health (SLOSH), which started in 2006 with a first follow-up of the participants of the Swedish Work Environment Survey 2003. Since then, additional participants of later Work Environment Surveys have been added. Ethical approval for SLOSH was obtained from the Regional Research Ethics Board in Stockholm (2010/0145-32, 2012/373-31/5, 2013/2173-32, 2015/2187-32). All participants gave their informed consent before taking part in the study.

**Fig. 1:**
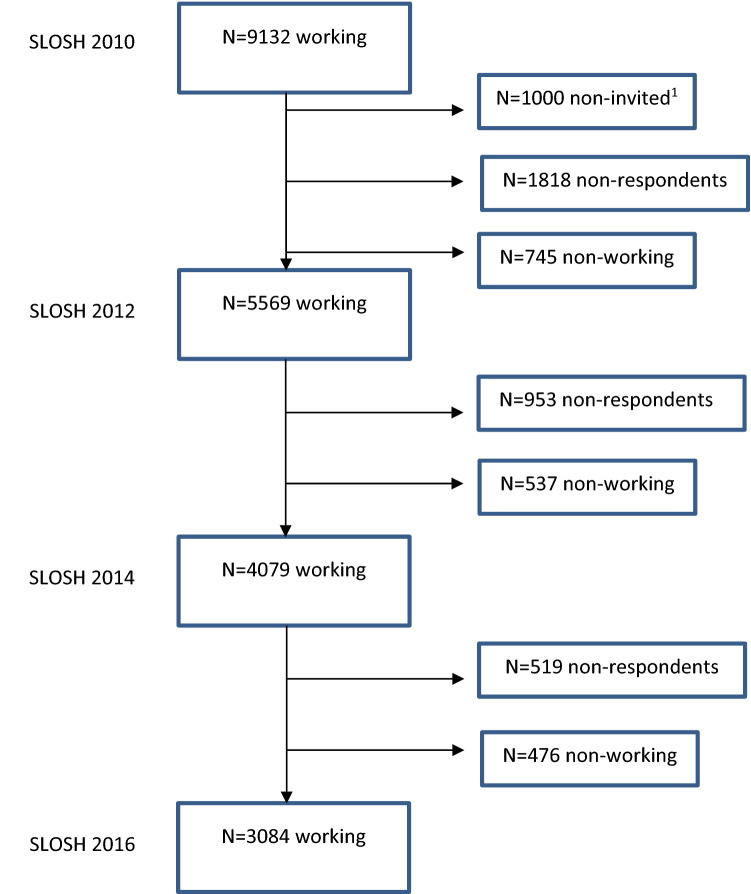
Number of participants over time. ^1^Because of a shortage in funding

The current study is based on data from four waves with response rates of 56.8% (*n* = 11,525) in 2010, 56.8% (*n* = 9880) in 2012, 52.2% (*n* = 20,216) in 2014, and 50.9% (*n* = 19,360) in 2016. The SLOSH data are approximately representative of the Swedish working population (http://www.slosh.se/; Magnusson Hanson et al. 2018). The study sample included participants who were employed for at least 30% of a fulltime job and who participated in all four waves (*n* = 3084), consisting of 58.1% women with a mean age of 48.31 years (SD = 8.10). See the flow chart in Fig. 1. To check for possible differences between the participants of the study sample and the participants in 2010 who were not included in the final study sample (dropout sample), we compared both samples on demographic variables (age, sex, fulltime employment, and education). We found significant but small differences between both samples (*η*^*2*^ < 0.06 and Cramer’s *V* ≤ 0.20). Participants of the study sample were, with a mean age of 48.31 years (*SD* = 8.10), somewhat younger than participants of the dropout sample (*M* = 50.48, SD = 11.03), *F*(1,9130) = 93.309, *p* = 0.000, *η*^*2*^ = 0.01 (small effect). Also, the study sample composed of relatively more women (58.1% vs. 54.6%; χ^2^(1) = 10.013, *p* = 0.002, Cramer’s *V* = 0.033, small effect) and more fulltime employed participants (82.2% vs. 77.2%, *χ*^*2*^(1) = 30.311, *p* = 0.000, Cramer’s *V* = 0.058, small effect), and the study sample was relatively higher educated (44.3% vs. 39.3% above upper secondary level; *χ*^*2*^(4) = 48.642, *p* = 0.000, Cramer’s *V* = 0.073, small effect). Given these small effect sizes, we deemed the study sample representative of the SLOSH sample in 2010.

### Measurements

#### Predictors

The predictor variables, involuntary and voluntary job change and reorganization, were measured at each wave and referred to the past two years.

Involuntary and voluntary job changes were measured with the single question *“If you changed jobs, was it voluntarily or not?”*, with four response options: (1) I have not changed jobs; (2) my job change was completely voluntary; (3) my job change was partly forced but I was not against it; and (4) my job change was against my will. The response options were combined into three categories (involuntary job change, voluntary job change, and no job change). These categories were coded as two dummy variables (with values 1 or 0). The first dummy, reflecting involuntary job change, was coded as 1 (and the second dummy as 0) when participants responded 3 or 4 on the original item. The second dummy, reflecting voluntary job change was coded as 1 (and the first dummy as 0) when participants responded a 2 on the original item. No job change was coded as 0 on both dummies.

Reorganizations were measured with the single question (Flovik et al. 2019; Stengård et al. 2017): *“Has a reorganization taken place at your workplace?”*, with four response options: (1) No, never (not relevant); (2) Yes, once, (3) = Yes, 2–3 times, (4) = Yes, 4 times or more. These response options were recoded as 0 when participants scored 1 on the original item. All other response options were coded as 1, indicating that participants had undergone a reorganization during the past two years.

#### Outcomes

The outcome variables, job and life satisfaction, and sleep disturbances, were measured at each wave. Job and life satisfaction were each measured with the single question: *“Overall, how satisfied are you with your work?”* and *“All things considered, how satisfied or dissatisfied are you with your life as a whole?”* Previous research (Nagy 2002) has supported the reliability of single-item satisfaction measures. Job satisfaction was measured using an 8-point scale with two anchors (1 = very dissatisfied and 8 = very satisfied). Life satisfaction was measured with seven response categories (1 = very dissatisfied, 2 = relatively dissatisfied, 3 = somewhat dissatisfied, 4 = neither one nor the other, 5 = somewhat satisfied, 6 = relatively satisfied, and 7 = very satisfied).

Sleep disturbances were measured with the validated four-item disturbed sleep index from the Karolinska Sleep Questionnaire (Nordin et al. 2013; Sacco et al. 2018). Participants were asked how often they had been disturbed in the previous 3 months by difficulties falling asleep, repeated awakenings with difficulties going back to sleep, premature (final) awakening, or disturbed or restless sleep. The response categories ranged from 1 (never) to 6 (always/five times a week). An average sleep disturbances score was calculated with a higher score indicating more sleep disturbances. Cronbach’s alphas for the four waves ranged from 0.84 to 0.85.

#### Moderators

Age and maladaptive coping expectations were included as moderator variables. Age was derived from registry data at the end of 2010 (the year of the first wave). Maladaptive coping expectations were assessed in the 2012 wave with six items from the validated TOMCATS questionnaire (i.e., the Theoretically Originated Measure of the Cognitive Activation Theory of Stress; Odéen et al. 2013; Ree et al. 2014) measuring maladaptive (hopelessness and helplessness) response–outcome expectancies. The response categories ranged from 1 (strongly disagree) to 4 (strongly agree). An example item is: *“I really do not have any control over the most important issues in my life.”* Cronbach’s alpha was 0.82.

#### Covariates

We included several covariates at the between-individuals and within-individuals level that may influence the outcome variables. As between-levels covariates, we included the demographic variables sex (0 = male, 1 = female) and education (1 = compulsory, 2 = 2-year upper secondary/vocational training, 3 = 3 or 4-year upper secondary, 4 = university or equivalent shorter than 3 years, 5 = university or equivalent 3 years or longer), both derived from registry data. As within-level covariates, we included time point (i.e., the four waves), full-time work (0 = part-time, 1 = full-time), and job demands that are known to impact (older) employees’ well-being. Job demands were assessed with five demands items of the Demands Control Questionnaire (Chungkham et al. 2013; Theorell 1996). Response categories ranged from 1 (often) to 4 (hardly ever/never). An example item is “*Do you have to work (very) fast?”* and four of the items were recoded so that a higher average score of the items indicated higher job demands. Cronbach’s alphas ranged from 0.71 to 0.74.

### Data analysis

As we were especially interested in intra-individual relationships over time and considering the nestedness of repeated measurements within individuals, we performed multilevel path modeling with ML estimation in Mplus 7.4 (Muthén and Muthén 1998). Intra-class correlations were between 0.10 and 0.68, which indicate that between 32 and 90% of the variance in our study variables were on the within-level (i.e., within individuals), justifying a multilevel approach.

As our main variables contained variance on the within-level (*n* = 12,336 possible data points) and between level (*n* = 3084 respondents), all main variables were modeled on the within and between level. Age, education, and maladaptive coping expectations were grand mean-centered, and job demands were person mean-centered (Aguinis et al. 2013). All other variables were modeled on both levels and were not centered.

**Fig. 2:**
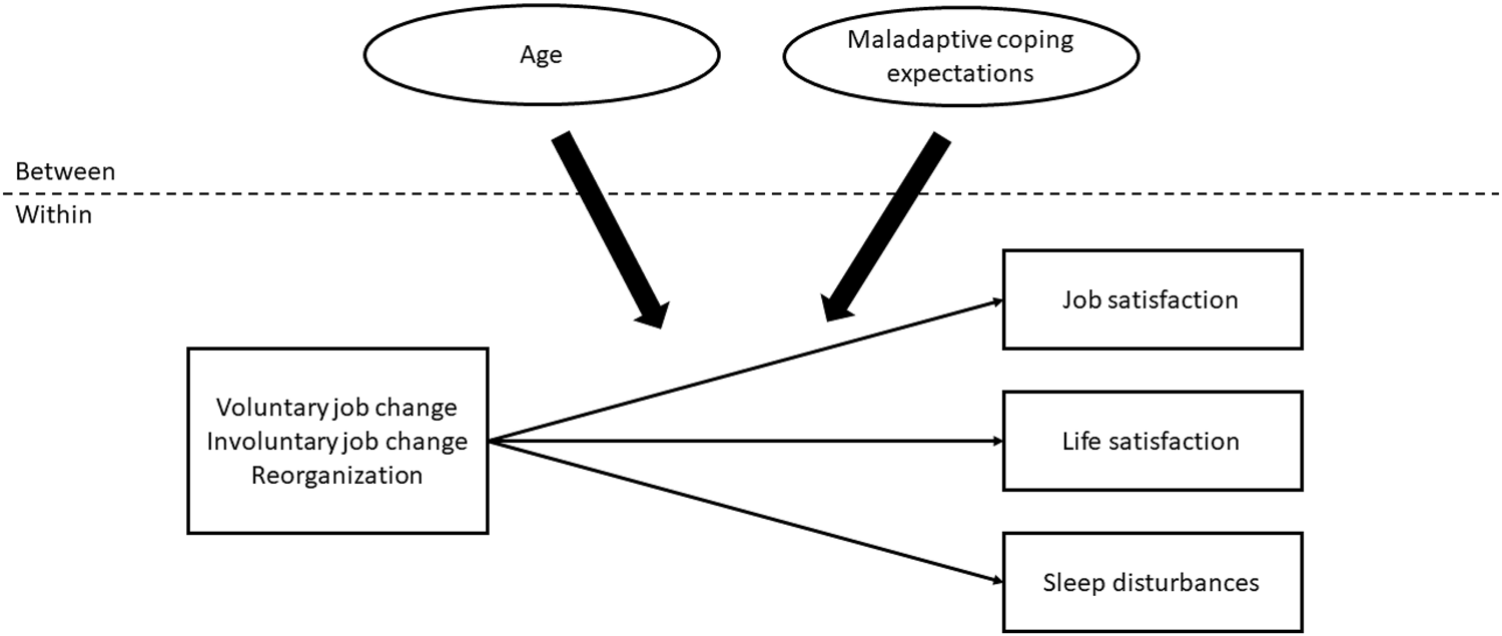
Multilevel path model

To examine the relationships among variables, we estimated two multilevel models. The first multilevel model (see Fig. 2) included data from all respondents. Pathways were specified from, respectively, the work change indicators to the outcomes, from covariates to the outcomes, and from age and maladaptive coping expectations to the outcomes. Next, cross-level interactions with age and maladaptive coping expectations were added.

The second multilevel model, which was almost identical to the first model, included respondents who were 50 years or older in 2010. We deemed this additional and exploratory analysis valuable to explore the moderating role of maladaptive coping expectations for older respondents. Hence, cross-level interactions were estimated for maladaptive coping expectations as cross-level moderator. Model fits were evaluated using the root mean square error of approximation (RMSEA) comparative fit index (CFI), and standardized root mean square residual (SRMR). Model fit is considered acceptable if RMSEA values are below 0.07, CFI values are above 0.95, and SRMR values are below 0.08 (Hu and Bentler 1999; Steiger 2007).

## Results

### Descriptive statistics

Means, standard deviations, intra-class correlations and between-levels and within-level correlations are presented in Table 1. The covariates (sex, education, full-time work, and job demands) were associated with several dependent and independent variables. Women reported more life satisfaction (*r* = 0.058, *p* < 0.003) and sleep disturbances (*r* = 0.136, *p* < 0.001) than men. Level of education was positively associated with voluntary job change (*r* = 0.247, *p* < 0.001) and reorganization (*r* = 0.099, *p* < 0.001), and fulltime employees more often experienced a voluntary job change (*r* = 0.119, *p* < 0.001) and reorganization (*r* = 0.182, *p* < 0.001) than part-time employees. As expected, higher job demands were associated with lower job satisfaction (between-levels: *r* = − 0.364, *p* < 0.001; within-level: *r* = − 0.205, *p* < 0.001) and life satisfaction (between-levels: *r* = − 0.216, *p* < 0.001; within-level: *r* = − 0.088, *p* < 0.001), and more sleep disturbances (between-levels: *r* = 0.347, *p* < 0.001; within-level: *r* = 0.142, *p* < 0.001). Of note is that age was negatively related to voluntary job change (*r* = − 0.367, *p* < 0.001) and reorganization (*r* = − 0.097, *p* < 0.001) but unrelated to involuntary job change (*r* = 0.020, *p* = 0.538). Further, age was not significantly related to maladaptive coping expectations (*r* =− 0.026, *p* = 0.151).

**Table 1:** Means, standard deviations, intra-class correlations, and inter-correlations among all study variables

	M	SD	ICC	1.	2.	3.	4.	5.	6.	7.	8.	9.	10.	11.
1. Sex: Female	58.1%			–										
2. Age	48.31	8.10		0.01	–									
3. Education	3.31	1.37		0.15***	− 0.14***	–								
4. Fulltime	81.1%			− 0.27***	− 0.13***	0.06**	–	0.07***	− 0.03*	− 0.02*	0.01	− 0.02	− 0.02*	0.04**
5. Job demands	2.61	0.45	0.60	0.09***	− 0.09***	0.10***	0.07***	–	− 0.08***	− 0.02	0.07***	− 0.21***	− 0.09***	0.14***
6. Voluntary job change	14.7%		0.14	0.07	− 0.37***	0.25***	0.12**	0.13***	−	− 0.07***	− 0.02	0.13***	0.03*	− 0.04***
7. Involuntary job change	5.7%		0.10	− 0.04	0.02	− 0.03	0.02	0.09*	0.05	−	0.04***	− 0.07***	− 0.04**	0.04***
8. Reorganization	51.6%		0.28	− 0.01	− 0.10***	0.10***	0.18***	0.27***	0.16***	0.19***	−	− 0.08***	− 0.02	0.02*
9. Job satisfaction	6.03	1.13	0.47	− 0.01	0.12***	− 0.01	− 0.05*	− 0.36***	− 0.09*	− 0.24***	− 0.19***	−	0.21***	− 0.16***
10. Life satisfaction	5.75	1.00	0.56	0.06**	0.08***	0.04	− 0.04*	− 0.22***	0.09**	− 0.19***	− 0.10***	0.62***	−	− 0.17***
11. Sleep disturbances	2.62	0.91	0.68	0.14***	0.09***	0.00	− 0.03	0.35***	− 0.06	0.13***	0.13***	− 0.38***	− 0.39***	−
12. Maladaptive coping expectations	1.38	0.39		− 0.03	− 0.03	− 0.06**	0.01	0.21***	− 0.09**	0.15***	0.08***	− 0.40***	− 0.55***	0.32***

*N* = 3084. Between-levels correlations are presented below the diagonal, within-level correlations are presented above the diagonal

*p < 0.05, **p < 0.01. ***p < 0.001

### Hypothesis testing

We tested whether job changes and reorganizations were related to job and life satisfaction and sleep disturbances, as moderated by age and maladaptive coping expectations. The multilevel model including all main paths fitted the data well, *χ*^*2*^ = 425.307, *df* = 21, RMSEA = 0.040, CFI = 0. 916, SRMR_within_ = 0.019, SRMR_between_ = 0.070. The results of this multilevel model are presented in Table 2. At the within-level of analysis, voluntary job change was positively related to job satisfaction (*γ* = 0.112, *p* < 0.001), negatively related to sleep disturbances (*γ* = − 0.027, *p* < 0.050), but not significantly related to life satisfaction (*γ* = 0.015, *p* = 0.182). In addition, involuntary job change was negatively related to job and life satisfaction (*γ* = − 0.066, *p* < 0.001; *γ* = − 0.037, *p* < 0.010) and positively related to sleep disturbances (γ = 0.045, *p* < 0.001), whereas reorganization was negatively related to job satisfaction (*γ* = − 0.058, *p* < 0.001) but unrelated to life satisfaction (*γ* = − 0.007, *p* = 0.537) and sleep disturbances (*γ* = 0.011, *p* = 0.286).

**Table 2:** Results from multilevel path analysis

	Job satisfaction			Life satisfaction			Sleep disturbances		
	γ	S.E.	*p* value	γ	S.E.	*p* value	γ	S.E.	*p* value
Within-level^1^									
Time	− 0.001	0.009	0.873	0.035	0.009	0.000	0.041	0.009	0.000
Fulltime	− 0.012	0.013	0.354	− 0.029	0.014	0.041	0.037	0.015	0.017
Job demands	− 0.166	0.009	0.000	− 0.074	0.009	0.000	0.121	0.009	0.000
Voluntary job change	0.112	0.011	0.000	0.015	0.011	0.182	− 0.027	0.011	0.013
Involuntary job change	− 0.066	0.011	0.000	− 0.037	0.011	0.001	0.045	0.011	0.000
Reorganization	− 0.058	0.010	0.000	− 0.007	0.011	0.537	0.011	0.011	0.286
Between-levels^1^									
Female Sex	− 0.022	0.019	0.248	0.034	0.017	0.054	0.149	0.018	0.000
Age	0.102	0.019	0.000	0.076	0.018	0.000	0.104	0.018	0.000
Education	− 0.010	0.019	0.601	0.006	0.018	0.745	0.010	0.018	0.605
Voluntary job change	− 0.036	0.034	0.300	0.100	0.031	0.001	− 0.029	0.032	0.364
Involuntary job change	− 0.153	0.037	0.000	− 0.101	0.034	0.003	0.072	0.035	0.040
Reorganization	− 0.114	0.027	0.000	− 0.047	0.025	0.060	0.102	0.025	0.000
Maladaptive coping expectations	− 0.374	0.018	0.000	− 0.537	0.015	0.000	0.313	0.017	0.000
Cross-level interactions^2^									
Age × VJC^3^	− 0.002	0.004	0.547	0.002	0.003	0.420	0.005	0.002	0.018
Age × IJC^4^	− 0.029	0.016	0.068	0.012	0.011	0.257	0.010	0.008	0.216
Age × REO^5^	0.005	0.002	0.062	0.002	0.002	0.270	0.008	0.002	0.000
MCE^6^ × VJC^3^	− 0.070	0.080	0.381	− 0.181	0.066	0.006	0.159	0.050	0.002
MCE^6^ × IJC^4^	0.422	0.250	0.091	− 0.211	0.176	0.232	0.287	0.128	0.025
MCE^6^ × REO^5^	− 0.089	0.053	0.095	− 0.449	0.041	0.000	0.071	0.033	0.032

Due to missing values, *N* ranges from 3067 to 3069

^1^Estimates reflect standardized coefficients

^2^Estimates reflect unstandardized coefficients

^3^Voluntary job change

^4^Involuntary job change

^5^Reorganization

^6^Maladaptive coping expectations

These results at the within-level of analysis differed in part from those at the between-levels. That is, at the between-levels of analysis, voluntary job change was positively related only to life satisfaction (*γ* = 0.100, *p* < 0.010), but unrelated to job satisfaction (*γ* = -0.036, *p* = 0.300) and sleep disturbances (*γ* = − 0.029, *p* = 0.364). The relationships of involuntary job change and reorganization with the outcome variables were largely the same, except that reorganization was positively related to sleep disturbances at the between level of analysis (*γ* = 0.102, *p* < 0.001) while no such relation was found at the within-level.

We explored possible age differences in the outcomes. Older employees reported higher job and life satisfaction (*γ* = 0.102, *p* = 0.000 and *γ* = 0.076, *p* = 0.000, respectively) and more sleep disturbances (*γ* = 0.104, *p* = 0.000) than younger employees. Furthermore, voluntary job change was related to less sleep disturbances in younger employees only (cross-level interaction: *γ* = 0.005, *p* < 0.050; + 1SD: γ = − 0.004, *p* = 0.885; − 1SD: *γ* = − 0.093, *p* = 0.000), while a reorganization was related to more sleep disturbances in older employees only (cross-level interaction: γ = 0.008, *p* < 0.001; + 1SD: *γ* = 0.102, *p* = 0.000; − 1SD: *γ* = − 0.029, *p* = 0.128). Age did not moderate any of the other relationships between work changes and outcomes.

We proposed that involuntary job change and reorganization would primarily harm the well-being of employees with maladaptive coping expectations. Maladaptive coping expectations were associated with lower job and life satisfaction (*γ* =− 0.374, *p* = 0.000 and *γ* = 0.537, *p* = 0.000, respectively) and more sleep disturbances (*γ* = 0.313, *p* = 0.000). Further, involuntary job change was only positively related to sleep disturbances among employees with more maladaptive coping expectations (cross-level interaction: *γ* = 0.287, *p* = 0.025; + 1SD: *γ* = 0.351, *p* = 0.000; − 1SD: *γ* = 0.126, *p* = 0.176). Also, reorganization was negatively related to the life satisfaction of employees with more maladaptive coping expectations while this relationship was positive for employees with less maladaptive coping expectations (*γ* =− 0.449, + 1SD: *γ* = − 0.202, *p* = 0.000; − 1SD: *γ* = 0.149, *p* = 0.000). Moreover, reorganization was related to sleep disturbances only in employees with more maladaptive coping expectations (*γ* = 0.071, *p* < 0.050; + 1SD: *γ* = 0.067, *p* = 0.001; − 1SD: *γ* = 0.011, *p* = 0.558). Further, there were significant cross-level interactions of voluntary job change and maladaptive coping expectations on life satisfaction (*γ* = − 0.181, *p* < 0.010; + 1SD: *γ* = 0.015, *p* = 0.687; − 1SD: *γ* = 0.156, *p* = 0. 0.000) and sleep disturbances (*γ* = 0.159, *p* < 0.010; + 1SD: *γ* = 0.011, *p* = 0.711; − 1SD: *γ* = − 0.114, *p* = 0.000): voluntary job change was associated with greater life satisfaction and less sleep disturbances in employees with less maladaptive coping expectations whereas there were no relationships between these variables in employees with more maladaptive coping expectations.

Finally, we explored if maladaptive coping expectations also moderated older employees’ responses to change. We estimated a multilevel path model with a subsample of older employees (*n* = 1497, 58.4% female, mean age = 55.12, SD = 3.54), which fitted the data well, *χ*^*2*^ = 122.751, *df* = 19, RMSEA = 0.031, CFI = 0.952, SRMR_within_ = 0.019, SRMR_between_ = 0.047. Table 3 shows that the within-level relationships between work changes and the outcome variables were largely the same as in the full sample, except that voluntary job change was unrelated to sleep disturbances (*γ* = − 0.001, *p* = 0.950). The between-levels relationships in this older sample were also largely the same as in the full sample, except that reorganization was now negatively related to life satisfaction (*γ* = − 0.073, *p* < 0.050).

**Table 3:** Results of the multilevel path analysis with a subsample of older workers

	Job satisfaction			Life satisfaction			Sleep disturbances		
	γ	S.E.	*p* value	γ	S.E.	*p* value	γ	S.E.	*p* value
Within-level^1^									
Time	0.006	0.013	0.672	0.069	0.013	0.000	0.031	0.013	0.018
Fulltime	− 0.033	0.020	0.097	− 0.009	0.021	0.681	0.030	0.023	0.195
Job demands	− 0.144	0.013	0.000	− 0.062	0.013	0.000	0.126	0.013	0.000
Voluntary job change	0.061	0.015	0.000	0.013	0.016	0.396	− 0.001	0.016	0.950
Involuntary job change	− 0.087	0.015	0.000	− 0.047	0.016	0.003	0.075	0.016	0.000
Reorganization	− 0.075	0.015	0.000	− 0.023	0.015	0.127	0.031	0.013	0.018
Between-levels^1^									
Sex	− 0.046	0.027	0.096	0.025	0.025	0.325	0.180	0.026	0.000
Education	− 0.025	0.027	0.355	− 0.039	0.025	0.120	0.024	0.026	0.350
Voluntary job change	− 0.027	0.046	0.556	0.091	0.041	0.027	− 0.024	0.042	0.565
Involuntary job change	− 0.162	0.046	0.000	− 0.105	0.042	0.012	0.105	0.043	0.014
Reorganization	− 0.159	0.037	0.000	− 0.073	0.034	0.030	0.113	0.035	0.001
Maladaptive coping expectations	− 0.372	0.026	0.000	− 0.527	0.021	0.000	0.299	0.025	0.000
Cross-level interactions^2^									
MCE^6^ × VJC^3^	− 0.014	0.134	0.919	− 0.032	0.101	0.749	0.097	0.078	0.213
MCE^6^ × IJC^4^	0.411	0.383	0.283	− 0.367	0.252	0.146	0.707	0.183	0.000
MCE^6^ × REO^5^	− 0.045	0.081	0.579	− 0.417	0.057	0.000	− 0.025	0.047	0.589

Due to missing values, *N* ranges from 1482 to 1483

^1^Estimates reflect standardized coefficients

^2^Estimates reflect unstandardized coefficients

^3^Voluntary job change

^4^Involuntary job change

^5^Reorganization

^6^Maladaptive coping expectations

Similar as in the full sample, reorganization was only negatively related to life satisfaction among older employees with more maladaptive coping expectations while this relationship was positive for older employees with less maladaptive coping expectations (cross-level interaction: *γ* = − 0.417, *p* < 0.001; + 1SD: *γ* = − 0.224, *p* = 0.000; − 1SD: *γ* = 0.101, *p* = 0.003). In addition, involuntary job change was only positively related to more sleep disturbances in older employees with more maladaptive coping expectations (cross-level interaction: *γ* = 0.707, *p* < 0.001; + 1SD: *γ* = 0.578, *p* = 0.000; − 1SD: *γ* = 0.028, *p* = 0.833).

## Discussion

We examined employee well-being outcomes (job and life satisfaction, and sleep disturbances) after work changes and explored whether these outcomes depended on employee age and maladaptive coping expectations. In line with prior research (Chadi and Hetschko 2018, 2021), we found that voluntary job changes were related to greater job satisfaction. Although voluntary job changes were also associated with life satisfaction when analyzed across employees, we could not confirm this relationship when analyzed at the within-person level. We extended prior research by also investigating associations between voluntary job changes and sleep disturbances. We found that these changes were associated with less sleep disturbances in younger employees and in employees with less maladaptive coping expectations. It should be noted that younger employees reported voluntary job changes more often than older employees.

In contrast to sparse previous research that could not establish negative effects of involuntary job changes (Chadi and Hetschko 2021; Equeter et al. 2018), we found that involuntary job changes were associated with lower job and life satisfaction, irrespective of employee age and maladaptive coping expectations. Moreover, involuntary job changes were associated with more sleep disturbances in workers with more maladaptive coping expectations.

Finally, corroborating research on employees’ adverse reactions to organizational reorganization (Bamberger et al. 2012; De Jong et al. 2016; Greubel and Kecklund 2011; Rafferty and Jimmieson 2017), we found that reorganizations were associated with lower job satisfaction. In addition, reorganizations were associated with more sleep disturbances among older employees and with lower life satisfaction and more sleep disturbances among employees with more maladaptive coping expectations. Also, we found that work changes were related to job satisfaction, irrespective of employee age and maladaptive coping expectations. The relationships between work changes and life satisfaction were less straightforward: involuntary but not voluntary job changes were related to life satisfaction, and reorganizations only related to lower life satisfaction in employees with more maladaptive coping expectations.

### Age and coping expectations

Both theory (Carstensen 2006; Kooij et al. 2011) and literature on aging at work (Boockmann et al. 2018; Niessen et al. 2010; Yeatts et al. 2000) deem it is plausible that older workers may be harmed more by job changes and reorganizations than younger workers, but to our knowledge, there is no empirical evidence supporting this contention. We, therefore, did not develop and test hypotheses (e.g., Rubin 2017)—and the assumptions that underlie them—about age differences in responses to work changes but rather explored these possible age differences. We found little evidence for the stereotypical idea that older workers suffer more from work changes than younger employees. Employee age did not moderate the relationships between work changes and job and life satisfaction. Only some age effects were found for sleep disturbances. Younger employees reported relatively less sleep disturbances after a voluntary job change while older employees reported more sleep disturbances in times of a reorganization. The generally greater number of sleep disturbances among older employees, as shown in this study, may not easily diminish under relatively positive conditions, such as a voluntary job change, while they may further increase during times of uncertainty (Lee et al. 2018; Palmer et al. 2017). Indeed, older employees experienced more sleep disturbances when experiencing reorganizations. Remarkably, while involuntary job changes were generally associated with lower job and life satisfaction, they were not associated with increased sleep disturbances.

We proposed that involuntary job changes and reorganizations would primarily harm the well-being of employees with maladaptive coping expectations. Indeed, we found that these employees reported lower life satisfaction and more sleep disturbances when having experienced an involuntary job change and more sleep disturbances after a reorganization than employees with less maladaptive coping expectations.

### Strengths and limitations

A major strength is that we used a four-wave study design with biennial survey data from the Swedish Longitudinal Occupational Survey of Health (SLOSH), which remained approximately representative of the Swedish working population after data selection. This design allowed us to examine relationships at the within-person level, which reduces Type II errors and confounds associated with individual differences. Our within-person and between-persons analyses show similar but also different results. While the relationships between involuntary job changes and the three outcome variables were comparable for both levels of analysis, the relationships between voluntary job changes and outcomes at the within-persons level were different from those at the between-persons level. We found, for example, a positive relationship between voluntary job changes and job satisfaction at the within-persons level but not at the between-persons level. Our within-persons findings suggest that people are relatively more satisfied with their jobs when they recently have experienced a voluntary job change than when they have not experienced a job change, whereas the between-persons findings tell us that voluntary job changers and job stayers have similar levels of job satisfaction. Obviously, results obtained from between-persons data should not be attributed to individuals (ecological fallacy) (Curran and Bauer 2011). Having said this, no causal inferences can be drawn from our within-persons findings, as the data are cross-sectional in nature at each measurement point. Also, our data on predictors, outcomes and covariates were self-reported. Therefore, future research could combine longitudinal objective data on work changes with longitudinal data on self-reported and—if possible—objective indicators of individual well-being (e.g., absenteeism, sick leave).

Other limitations of this study concern the study sample and some of the measures. Although the SLOSH sample approximately represents the Swedish working population and our selected study sample did not substantially differ from the drop-out, prior researches (Magnusson Hanson et al. 2018) as well as this study have shown that women and higher educated employees are somewhat overrepresented in the data. Moreover, our findings are based on a Swedish sample. This means that our findings may be less generalizable to other, for example non-Western, countries with different work and economic standards, work practices and conditions, and attitudes toward aging (North and Fiske 2015). It would be valuable if future research would take a lifespan developmental perspective (Baltes et al 1980), which considers the sociocultural and historical context of individuals, and investigate the role of cultural differences for the relationships between work changes and outcomes of younger and older workers. All in all, we should be careful in making firm statements about the generalizability of our results.

In addition, some of our constructs were measured with a single question, which means that we could not estimate the psychometric properties of these measures. Single-item measures are acceptable for concrete and inclusive constructs (Fisher et al. 2016; Fuchs and Diamantopoulos 2009), such as job and life satisfaction. Prior research has supported the reliability of our single-item measures of job and life satisfaction (Fisher et al. 2016; Nagy 2002). We are, therefore, confident that our measures of job and life satisfaction adequately reflect the constructs. While study participants will also be well able to respond to the single-item measure of whether they have undergone a reorganization at their own workplace (yes/no) and this item has been used in other research (Fløvik et al. 2019; Stengård et al. 2017), our measure provides no information on the precise nature and amount of the reorganization. Fløvik et al. (2019) used several single-item measures to assess different types of organizational changes (reorganization, downsizing, lay-offs, partial company closure, partial company outsourcing, and change of company ownership/acquisition) and found that only reorganization, downsizing, and layoffs were significantly related to mental distress. Our results correspond with these and other findings (e.g., Rafferty and Jimmieson 2017) of the detrimental effects of organizational changes at the workplace. Yet, we are unable to disentangle which aspects of a reorganization may cause these effects. Further research could use a more extended reorganization measure as to gain a better understanding of how older and younger employees experience different types of reorganizations.

### Theoretical and practical implications

The findings of this study have some theoretical implications. First, both Job Demand-Resources (JD-R) theory (Bakker and Demerouti 2017) and Conservation of Resources Theory (COR) (Hobfoll et al. 2018) argue that individuals’ well-being depend on their resources and the ability to allocate these resources. Job resources (e.g., autonomy, organizational support) and personal resources (e.g., resilience) positively affect people’s well-being directly and they buffer the negative effects of high demands. In this study, we found that employees who had experienced a reorganization reported higher job demands than those who had not. Further, we evidenced that maladaptive coping expectations were directly related to well-being outcomes and moderated most of the relationships between reorganizations and well-being outcomes. All in all, these findings support COR and JD-R theories and highlight the importance of building and retaining personal resources such as adaptive coping expectations. While job and personal resources are often treated as simultaneously and independently affecting employee well-being, some research has shown that these resources are reversely and causally related (Bakker and Demerouti 2017). This is consistent with COR theory that denotes the existence of so-called gain (and loss) spirals: an increase (or decrease) in resources will lead to an increase (or decrease) in other resources. Future research could investigate (reversal) relationships between employees’ job resources and adaptive coping expectations, before, during and after reorganizations.

Second, we did not find strong support for Selection, Optimization, and Compensation (SOC) theory (Baltes et al. 1999), which argues that older people have stronger maintenance and security motives than younger ones and thus will have more problems with adapting to, for example, involuntary job changes. In this study, we did not find age differences in well-being after involuntary changes. This could mean that older workers did not have maintenance motives to a greater extent than younger workers. However, an alternative explanation is that older workers had stronger maintenance motives than younger ones but were yet well able to adapt to situations that defy these motives. In other words, we do not know whether older and younger workers set and prioritize similar goals to achieve a desired state (selection) or that older workers allocate extra resources, such as time and effort (optimization), or new or unused resources (compensation) to cope with involuntary changes. SOC strategy use seems positively but weakly related to age (Moghimi et al. 2017). To extend our knowledge of adaptation processes in younger and older workers, future research could examine age differences in SOC strategies in times of work changes.

This study also provides practical implications for employers and policy makers. Involuntary job changes and reorganizations can undermine the job satisfaction of both younger and older workers. Low job satisfaction is associated with employee turnover (Wright and Bonett 2007) and the intentions of older employees to retire earlier (Davies et al. 2017; Zacher and Rudolph 2017). For retaining employees in times of organizational changes, organizations could pay particular attention to their justice climate in general and the perceived fairness of change procedures in particular. Employee organizational justice perceptions are associated with employee health (Herr et al. 2018), and individual and group-level outcomes, such as organizational commitment and turnover intentions (Simons and Roberson 2003), and retirement decisions (Eib et al. 2021). Organizations that create a climate in which resources are fairly distributed, rules for decision-making are transparent and fair, and social interactions are informative and respectful, are better able to reduce negative responses to a reorganization and retain committed employees than organizations that thwart employees’ need for fairness (Kernan and Hanges 2002). Stereotyping and making an unwarranted distinction between younger and older employees are hallmarks of unfairness and should therefore be avoided. Organizational leaders who believe that older and younger workers should be treated equally and who are positive toward late retirement foster an organization’s actual recruitment and retention of older workers (Oude Mulders et al. 2017). These age-inclusive practices, in turn, promote employees’ social exchange perceptions and reduce turnover intentions (Boehm et al. 2014).

While it is important to retain older workers in the labor market, there seems no need to accommodate them or treat them with special care in times of organizational changes. On the contrary, specific measures for older workers may highlight the persistent stereotype that aging is associated with inflexibility, less adaptiveness and work engagement. Older workers may encounter ageism and internalize negative age stereotypes, which may reduce their sense of belonging in the workplace and, consequently, their work motivation (Thorsen et al. 2012; Rahn et al. 2021). Instead, it may be better if organizations continue to provide older workers with activating workdays (with high work pressure and autonomy), which will promote their work engagement and performance (Kooij et al. 2020). Also, organizations could apply an HR policy that emphasizes older workers’ participation in training rather than their phasing out (e.g., lighter workload, semi-retirement), or demotion (movement to a less demanding position) as only training was found to promote job satisfaction (Visser et al. 2021).

Finally, organizations could develop interventions aiming at raising awareness of and preparation for change for all employees and particularly for those with maladaptive coping expectations. Interventions that foster employees’ adaptive responses to future career transitions may be particularly useful here (Van der Horst and Klehe 2019). Further, organizations could involve their employees in implementing the changes, increasing their expectation of success (Nielsen 2013).

## Concluding remarks

The findings of this study provide evidence that involuntary job changes and reorganizations are associated with lower employee well-being. Employees with maladaptive coping expectations in particular are prone to suffer from sleep disturbances when experiencing these changes. Contrary to extant stereotypic beliefs about older workers, we found little evidence that older workers respond more negatively to work changes than younger ones. Future research could substantiate our findings by incorporating objective measures in a longitudinal study design. For now, we advise organizations to maintain the work engagement of older workers by treating them like younger workers.

## Data Availability

Given restrictions from the ethical review board and considering that sensitive personal data are handled, it is not possible to make the data freely available. Access to the data may be provided to other researchers in line with Swedish law and after consultation with the Stockholm University legal department. Requests for data should be sent to registrator@su.se with reference to ‘SLOSH-Work changes and employee age, maladaptive coping expectations, and well-being’ or directly to the corresponding author.
